# Soluble Ligands for the NKG2D Receptor Are Released during Endometriosis and Correlate with Disease Severity

**DOI:** 10.1371/journal.pone.0119961

**Published:** 2015-03-16

**Authors:** Iñaki González-Foruria, Pietro Santulli, Sandrine Chouzenoux, Francisco Carmona, Frédéric Batteux, Charles Chapron

**Affiliations:** 1 Département Développement, Reproduction et Cancer, Institut Cochin, INSERM, Paris, France; 2 Université Paris Descartes, Sorbone Paris Cité, Faculté de Médecine, Assistance Publique—Hôpitaux de Paris (AP-HP), Groupe Hospitalier Universitaire (GHU) Ouest, Centre Hospitalier Universitaire (CHU) Cochin, Department of Gynecology Obstetrics II and Reproductive Medicine, Paris, France; 3 Institut Clinic of Gynecology, Obstetrics and Neonatology, Hospital Clínic-Institut d'Investigacions Biomèdiques August Pi i Sunyer (IDIBAPS), Faculty of Medicine-University of Barcelona, Barcelona, Spain; 4 Service d’immunologie biologique, Hôpital Cochin, Paris cedex 14, France; 5 DHU Risque et grossesse, Hôpital Cochin, Paris cedex 14, France; University of Edinburgh, UNITED KINGDOM

## Abstract

**Background:**

Endometriosis is a benign gynaecological disease. Abundant bulk of evidence suggests that patients with endometriosis have an immunity dysfunction that enables ectopic endometrial cells to implant and proliferate. Previous studies show that natural killer cells have a pivotal role in the immune control of endometriosis.

**Methods and Findings:**

This is a prospective laboratory study conducted in a tertiary-care university hospital between January 2011 and April 2013. We investigated non-pregnant, younger than 42-year-old patients (*n*= 202) during surgery for benign gynaecological conditions. After complete surgical exploration of the abdominopelvic cavity, 121 women with histologically proven endometriosis and 81 endometriosis-free controls women were enrolled. Patients with endometriosis were classified according to a surgical classification in three different types of endometriosis: superficial peritoneal endometriosis (SUP), ovarian endometrioma (OMA) and deep infiltrating endometriosis (DIE). Peritoneal fluid samples were obtained from all study participants during the surgery in order to detect soluble NKG2D ligands (MICA, MICB and ULBP-2). When samples with undetectable peritoneal fluid levels of MICA, MICB and ULBP-2 were excluded, MICA ratio levels were significantly higher in endometriosis patients than in controls (median, 1.1 pg/mg; range, 0.1–143.5 versus median, 0.6 pg/mg; range, 0.1–3.5; p=0.003). In a similar manner peritoneal fluid MICB levels were also increased in endometriosis-affected patients compared with disease-free women (median, 4.6 pg/mg; range, 1.2–4702 versus median, 3.4 pg/mg; range, 0.7–20.1; p=0.001). According to the surgical classification, peritoneal fluid soluble MICA, MICB and ULBP-2 ratio levels were significantly increased in DIE as compared to controls (p=0.015, p=0.003 and p=0.045 respectively). MICA ratio levels also correlated with dysmenorrhea (r=0.232; p=0.029), total rAFS score (r=0.221; p=0.031) and adhesions rAFS score (r=0.221; p=0.031).

**Conclusions:**

We demonstrate a significant increase of peritoneal fluid NKG2D ligands in women with endometriosis especially in those cases presenting DIE. This study suggests that NKG2D ligands shedding is a novel pathway in endometriosis complex pathogenesis that impairs NK cell function.

## Introduction

Endometriosis is a benign chronic gynaecological disease characterized by the presence of endometrium-like tissue-glands and stroma- outside the uterine cavity [1]. This condition may affect up to 10–15% of women in childbearing-age, causing pelvic pain [2], and infertility [3].

Although endometriosis was first reported by Carl von Rokitansky more than a hundred years ago [4], the pathogenesis of this condition is still not clear [5]. Sampson's theory of retrograde menstruation is probably the most accepted among scientific community, though this explanation cannot adequately account for all the pathogenesis of the disease [6]. Previous studies report that retrograde menstruation occurs in >90% of women [7], nevertheless, the incidence of endometriosis is much lower in general population, which means that there should be physiological mechanisms with scavenging capacity that are able to eliminate the ectopic endometrial implants from the menstrual reflux.

Abundant bulk of evidence suggests that patients with endometriosis have an immunity dysfunction that enables ectopic endometrial cells to implant and proliferate [8–11]. Notwithstanding, the onset and progression of the disease is probably not only an immune issue, but the result of a complex series of processes that lead to the attachment of endometrial cells to the peritoneal surface [12], invasion and estrogen-related proliferation [13], vasculogenesis [14], angiogenesis and finally chronic inflammation [15,16]. Chronic inflammation is associated with an overproduction of prostaglandins [17], metalloproteinases, cytokines and chemokines, creating a self-supporting loop that mantains and amplificates the progression of the disease [18–20]. Once the process is started, many profibrotic mediators also play a role in the fibrogenesis associated with endometriosis [21].

From an immunological point of view, it has been shown that circulating natural killer (NK) cells are capable of destroying endometrial cells [22]. Thus, it has been proposed that NK cells may have a pivotal role in the immune control of endometriosis [23,24]. Additionally, many studies demonstrate that NK cells in blood and peritoneal fluid of endometriosis patients present a decreased cytotoxicity to autologous and heterologous endometrium [25,26].

The activity of NK cells is regulated through the signals from their receptors in a highly complex manner. NKG2D is an activating C-type lectin-like NK cell receptor involved in the elimination of infected or transformed cells. Upon binding to the corresponding ligands, NKG2D triggers a cytotoxic response that activates NK cells.

There have been described multiple ligands for this receptor (NKG2DLs), which are members of the *major histocompatibility complex* (MHC) class I family. In humans these ligands are MHC class-I chain related proteins (MIC) A, MICB and UL16 binding protein (ULBP) 1–6 [27–29]. Paradoxically, the presence of the soluble form of NKG2DLs generates an inhibitory action on NK cells. In fact, it has been shown that increased levels of soluble NKG2DLs are present in the sera of cancer patients and this mechanism has been proposed as a strategy for tumours to avoid immune surveillance [30,31].

Research in the mechanisms that lead to the immune dysfunction present in endometriosis is a key step to better understand the pathogenesis of the disease.

The aim of the present study is to find a novel pathway that impairs NK cell function and facilitates the implantation and growth of refluxed endometrial cells over the peritoneal surface. For this reason, we assayed peritoneal fluid NKG2DLs (MICA, MICB and ULBP-2) obtained from patients with histologically proven endometriosis and endometriosis-free women. These results were compared and correlated among them and with respect of the endometriosis phenotype and the severity of the disease.

## Materials and Methods

### Patients

The local ethics committee (CCPPRB: Comité Consultatif de Protection des Personnes dans la Recherche Biomédicale) of Paris Cochin approved the study protocol and all study participants gave informed written consent for the enrolment in the study protocol.

From January 2011 to April 2013, a continuous series of 202 women, younger than 42-years-old, who underwent a laparoscopy for gynaecological reasons in our centre had been recruited in this study. Clinical and biological data were prospectively collected. Excluded from this population were pregnant women, women with cancer and those who did not give consent to the study. Women were classified into two groups depending on laparoscopic findings [32,33]: the endometriosis group consisted of subjects with histologically proven endometriosis, while patients in the control group did not show any macroscopic sign of the disease after a meticulous exploration of the abdominal cavity during the surgical procedure.

Endometriosis was staged and scored (total, implant and adhesion scores) according to the revised American Fertility Society (rAFS) Classification [34]. Besides, patients with endometriosis were also staged according to their endometriosis phenotype. Based on histological findings, endometriotic lesions were classified into three phenotypes: peritoneal superficial endometriosis (SUP), ovarian endometrioma (OMA) and deep infiltrating endometriosis (DIE). As these three types of lesions are frequently associated and may coexist [35] patients with endometriosis were classified according to the most severe finding. Endometriotic lesions are usually ranked from the least severe to the most severe in SUP, OMA and DIE [36]. By definition, DIE patients were graded from the least to the most severe DIE lesion as follows: uterosacral ligament (s), vagina, bladder, intestine and ureter [37]. The patient’s most severe localization was considered for grading.

The study analysis used a prospective managed database. For each patient, personal history data were obtained during face-to-face interviews, which were conducted by the surgeon the month before surgery. A highly structured previously published questionnaire was used for all patients [38,39]. The following items were recorded: age, parity, gravidity, height, weight, BMI, past history of hormonal and/or surgical treatment for endometriosis, existence of gynaecological pain symptoms (dysmenorrhea, deep dyspareunia, non-cyclic chronic pelvic pain—NCCPP-), gastrointestinal [40] and lower urinary tract symptoms. According to a previous publication, NCCPP is defined as intermittent or permanent pelvic pain not related to the menstrual cycle [41]. In order to evaluate the pain intensity preoperatively a previously validated 10-cm visual analog scale was used [42].

### Collection of peritoneal fluid samples

Peritoneal fluid was taken during surgery from all the patients of the study (202). The samples were centrifuged at 800 g for 10 min at 4°C and supernatants were collected. Aliquots of the samples were stored at -80°C until needed for the analysis.

### Measurement of NKG2D ligands concentration

MICA, MICB and ULBP-2 were assayed in the peritoneal fluid by an enzyme-linked immunosorbent assay (ELISA) (R&D Systems, Inc., Minneapolis, MN, USA), according to the manufacturer’s recommendations. The range of determination for MICA and ULBP-2 was 62’5–4.000 pg/ml and for MICB 156–10.000 pg/ml. The results below the lower threshold levels were considered as 0 pg/ml for the statistical analysis. Each sample was tested in duplicate and reflected the mean of the two measurements

Peritoneal fluid protein concentration was measured in all the samples using the spectroscopic Bradford protein assay method [43].

In order to avoid biases owing to peritoneal fluid concentration or dilution at the moment of obtaining the sample, a ratio between the NKG2D ligands result (pg/ml) and the protein concentration (mg/ml) was calculated for each peritoneal sample individually (values are expressed in pg of NKG2D ligand/ mg of protein).

### Statistical analysis

All data were collected in a computerized database and subsequently analyzed by Statistical Package for the Social Sciences Software (SPSS Inc., Chicago, IL, USA). When endometriosis and control samples were analyzed, Student’s t-test was used for quantitative variables and Pearson’s X2 or Fisher’s exact test were performed for qualitative variables. Considering the non-Gaussian distribution of MICA, MICB and ULBP-2 levels, statistical analysis between the two groups was performed with the Mann-Whitney U test.

When more than two groups were compared, Kruskal-Wallis test was used. When group medians were significantly different by the Kruskal-Wallis test (p<0,05), pairwise comparisons were performed using the with Dunn's Multiple Comparison Test.

In the view of the number of samples with undetectable levels of MICA, MICB and ULBP-2, two different statistical analyses were performed, one including and the other excluding the samples with undetectable levels of these NKG2D ligands. Correlations between MICA, MICB and ULBP-2 levels in peritoneal fluid and surgical findings and clinical characteristics of disease severity, measured with semiquantitative variables, were examined using the non-parametric Spearman’s rank correlation test. P<0,05 was considered statistically significant.

## Results

### Study Population

One hundred and twenty one endometriosis-affected women and 81 disease free women were recruited for this study. Their clinical and surgical characteristics are displayed in Table 1.

**Table 1:** Baseline characteristics of participants.

Patient characteristics	Endometriosis (N = 121)	Controls (N = 81)	P
Age (years) ^a^	30.8 ± 5.1	31.7± 5.36	0.267^t^
Height (cm) ^a^	167.5 ± 6.1	164.1 ± 6.0	<0.001^t^
Weight (kg) ^a^	59.5 ± 8.1	61.5 ± 9.9	0.162^t^
BMI (kg/m^2^) ^a^	21.2 ± 2.5	22.8 ± 3.4	0.001^t^
Parity ^a^	0.2 ± 0.5	0.4 ± 0.7	0.093^t^
Gravidity ^a^	0.4 ± 0.7	0.6 ± 1.0	0.123^t^
Preoperative hormonal treatment (n, %)	48 (40.0%)	33 (40.1%)	0.887^k^
Infertility (n, %)	39 (32.2%)	30 (37.0%)	0.479^k^
Duration (month) ^a^	39.0 ± 30.1	30.0 ± 17.6	
Previous treatment for endometriosis:			
* hormonal treatment (n, %)*	68 (56.2%)	NA	
* previous surgery (n, %):	19 (15.7%)	NA	
* previous endometrioma’s surgery (n, %)	11 (9.1%)	NA	
Preoperative painful symptoms scores: ^a^ ^,^ ^b^ ^,^ ^c^			
Dysmenorrhea	6.3 ± 2.9	4.6 ± 2.9	<0.001^t^
Deep dyspareunia ^$^	4.1 ± 3.3	2.4 ± 3.0	0.001^t^
Non-cyclic chronic pelvic pain	2.9 ± 3.2	1.7 ± 2.6	0.011^t^
Gastrointestinal symptoms	3.2 ± 3.3	0.9 ± 1.9	<0.001^t^
Lower urinary symptoms	1.4 ± 2.8	0.2 ± 0.9	<0.001^t^
rAFS Classification:			
Mean implants score rAFS ^a^ ^,^ ^d^	11.3 ± 11.2	NA	
Mean adhesions score rAFS ^a^ ^,^ ^d^	9.6 ± 16.3	NA	
Mean total score rAFS ^a^ ^,^ ^d^	21.2 ± 23.1	NA	
rAFS stage (n, %): ^d^		NA	
I	37 (30.6%)		
II	16 (13.2%)		
III	26 (21.5%)		
IV	42 (34.7%)		
Surgical classification:			
Superficial endometriosis (n, %)	41 (33.9%)	NA	
Endometrioma (n,%)	32 (26.4%)	NA	
Endometrioma size (cm): ^a^		NA	
Right	4.9 ± 2.8		
Left	4.8 ± 3.1		
Endometrioma laterality (n, %):		NA	
Bilateral	7/32 (21.9%)		
Right	11/32 (34.4%)		
Left	14/32 (43.7%)		
DIE lesions (n, %) ^e^	48 (39.7%)	NA	
Mean number of DIE lesions ^a^	2.3 ± 1.5	NA	
Total number of DIE lesions (n, %):		NA	
1	13/48 (27.1%)		
2	11/48 (22.9%)		
≥3	24/48 (50.0%)		
Anatomical distribution of DIE (n, %): ^e^ ^,^ ^b^		NA	
USL	35/48 (72.9%)		
Vagina	15/48 (31.2%)		
Bladder	7/48 (14.6%)		
Intestine	23/48 (47.9%)		
Ureter	2/48 (4.2%)		
Worst DIE lesion (n, %): ^e^		NA	
USL	13/48 (27.1%)		
Vagina	7/48 (14.6%)		
Bladder	5/48 (10.4%)		
Intestine	21/48 (43.7%)		
Ureter	2/48 (4.2%)		

^a^ Data are presented as mean ± SD;

^b^ Sometimes more than one for the same patient;

^c^ Visual analogue scale (VAS);

^d^ Score according to the American Fertility Society Classification (34)

^e^ According to a previously published surgical classification for deeply infiltrating endometriosis (DIE) (37)

^t^ Student’s t-test;

^k^ Pearson’s chi-square test;

^$^ 4% of patients have no sexual intercourse at the moment of the surgery.

* <5% missing data

NA: not applicable

USL: uterosacral ligaments.

Following the endometriosis surgical classification [38,39] and based on the location of the worst lesion presented, the 121 histologically proven endometriotic patients were classified as follows: 41 (33.9%) SUP, 32 (26.4%) OMA (right 11; left 14; bilateral 7) and 48 (39.7%) DIE. Patients' distribution according to the worst lesion of DIE founded was the following: 13 (27.1%) uterosacral ligament(s), 7 (14.6%) vagina, 5 (10.4%) bladder, 21 (43.7%) intestine and 2 (4.2%) ureter. These 48 DIE patients presented a total of 113 histologically proven DIE lesions distributed as follows: 46 uterosacral ligament lesions, 15 vaginal lesions, 7 bladder lesions, 43 intestinal lesions (1 intestinal lesion in 10 patients and more than 1 intestinal lesion in 13 patients) and 2 ureteral lesions. The mean (± SD) number of DIE lesions per patient was 2.3 ± 1.5 (range 1–6).

The indications for surgery in the 81 endometriosis-free women recruited in the study were the following: uterine fibroids (29 patients, 35.8%), non-endometriotic benign ovarian cysts (21 patients, 25.9%), tubal infertility (15 patients, 18.6%), pelvic pain (7 patients, 8.6%) and other indications (9 patients, 11.1%).

There were no differences in age, gravidity, parity and infertility between the endometriosis group and control group. Body mass index (BMI) was significantly lower in endometriotic patients than controls (P = 0.001). The percentage of patients with preoperative hormonal treatment was similar between the two groups (Table 1).

### MICA, MICB and ULBP-2 levels

MICA, MICB and ULBP-2 levels were measured in peritoneal fluid of all the patients included in the study (n = 202). MICA, MICB and ULBP-2 were detected in the peritoneal fluid of 140 (69.3%), 196 (97.0%) and 34 (16.8%) study participants respectively.

MICA was detected in 82 (67.8%) endometriosis-affected women and in 58 (71.6%) controls (P = 0.641). MICB resulted positive in 118 (97.5%) patients with endometriosis and in 78 (96.3%) endometriosis-free patients (P = 0.685). Detection rate for ULPB-2 was significantly higher in endometriotic women as compared to controls (27 (22.3%) vs. 7 (8.6%), respectively; P = 0.012) (S1 Table).

According to the surgical classification, among endometriosis-affected women, MICA was detected in 25 (60.9%) SUP, 24 (75.0%) OMA and 33 (68.7%) DIE patients (P = 0.566). MICB became positive in 39 (95.1%) SUP, 31 (96.8%) OMA and 48 (100%) DIE patients (P = 0.543), while ULBP-2 was detected in 7 (17.1%) SUP, 10 (31.2%) OMA, 10 (20.8%) DIE patients (P = 0.026) (S1 Table).

**Fig 1:**
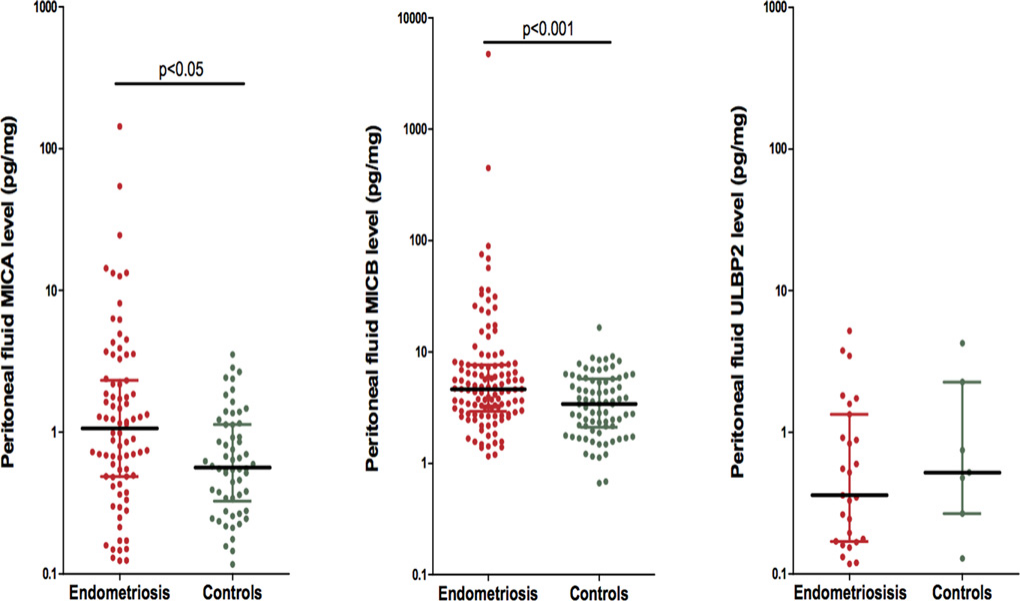
Peritoneal NKG2D ligands levels measured by ELISA in patients with endometriosis and in controls after exploration of the abdominopelvic cavity. Samples with undetectable levels of NKG2D ligands have been excluded. The peritoneal fluid MICA and MICB ratio levels among groups was significantly different by the Mann Whitney test (P = 0.035 and P<0.001 respectively). Peritoneal NKG2D ligands ratio values are represented on a logarithmic scale as a scatter dot plot. The medians with their interquartile range are reported.

When samples with undetectable peritoneal fluid levels of MICA, MICB and ULBP-2 were excluded, MICA and MICB levels were significantly higher in endometriosis patients than in controls (median, 1.1 pg/mg; range, 0.1–143.5 vs. median, 0.6 pg/mg; range, 0.1–3.5; P = 0.003 for MICA; median, 4.6 pg/mg; range, 1.2–4702 vs. median, 3.4 pg/mg; range, 0.7–20.1; P = 0.001 for MICB). In contrast, ULPB-2 levels in endometriosis patients were not significantly different from controls (median, 0.4 pg/mg; range, 0.1–5.2 vs. median, 0.5 pg/mg; range, 0.1–4.2; P = 0.551). In a similar way, when considering all the samples of endometriosis patients and controls, the levels of MICA (median, 0.5 pg/mg; range, 0.0–143.5 vs. median, 0.2 pg/mg; range, 0.0–3.5; P = 0.060), MICB (median, 4.9 pg/mg; range, 0.0–4702 vs. median, 3.4 pg/mg; range, 0.0–20.1; P< 0.001) and ULBP-2 (median, 0.0 pg/mg; range, 0.0–5.2 vs median, 0.0 pg/mg; range, 0.0–4.2; P = 0.014) resulted higher in the endometriosis group than in the control group, although for MICA the differences did not reach statistical significance. (Table 2 and Fig. 1).

**Table 2:** Statistical analyses for peritoneal NKG2D ligands (MICA, MICB and ULBP-2) ratio levels in women with endometriosis and controls.

xTable 2: NKG2D Ligands ratio levels in women with endometriosis and controls.			
	Endometriosis	Controls	p
Peritoneal MICA (with)	(n = 121) 0.48 (0.0–143.5)	(n = 81) 0.23 (0.0–3.5)	0.060 ^u^
Peritoneal MICA (without)	(n = 82) 1.06 (0.1–143.5)	(n = 58) 0.56 (0.1–3.5)	0.003 ^u^
Peritoneal MICB (with)	(n = 121) 4.87 (0.0–4702)	(n = 81) 3.40 (0.0–20.1)	<0.001 ^u^
Peritoneal MICB (without)	(n = 118) 4.61 (1.2–4702)	(n = 78) 3.48 (0.7–20.1)	0.001 ^u^
Peritoneal ULBP-2 (with)	(n = 121) 0.00 (0.0–5.2)	(n = 81) 0.00 (0.0–4.2)	0.014 ^u^
Peritoneal ULBP-2 (without)	(n = 27) 0.36 (0.1–5.2)	(n = 7) 0.52 (0.1–4.2)	0.551 ^u^

Analyses were performed both including samples with undetectable levels of NLG2D ligands (i.e. below lower limit of detection of the assay) and without these samples.

Results are expressed as median (range)

Values are expressed in pg/mg

u. Statistical analysis was performed with the Mann-Whitney U Test.

**Fig 2:**
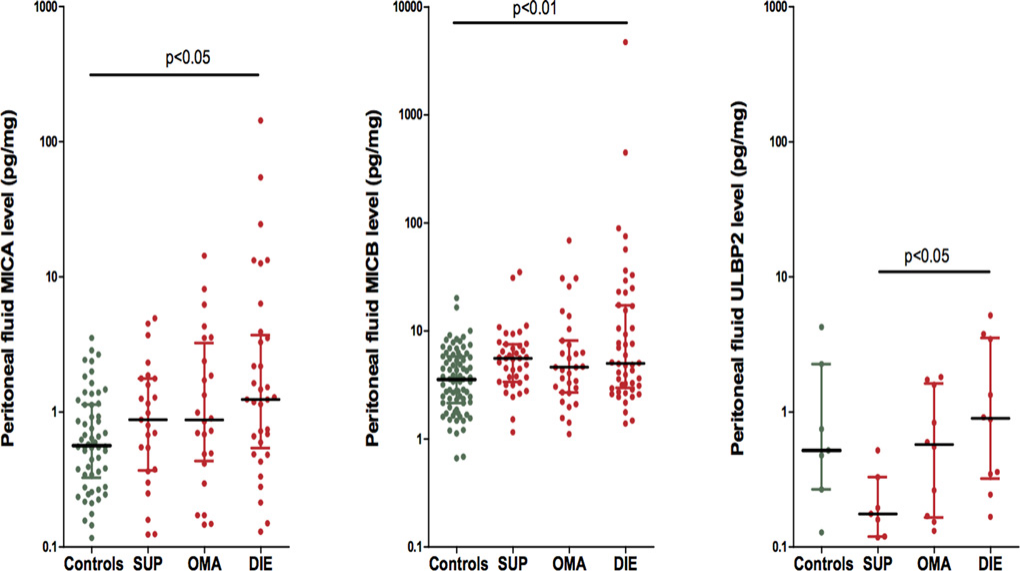
Peritoneal NKG2D ligands levels measured by ELISA in patients with endometriosis and in controls according to the surgical classification of endometriosis. Samples with undetectable levels of NKG2D ligands have been excluded. The peritoneal fluid MICA and MICB ratio levels among groups [DIE (n = 48), OMA (n = 31); SUP (n = 39) and controls (n = 78)] was significantly different by the Kruskal-Wallis test (P = 0.015 and P = 0.002 respectively). Post hoc test were performed using the with Dunn's Multiple Comparison Test. Peritoneal NKG2D ligands ratio values are represented on a logarithmic scale as a scatter dot plot. The medians with their interquartile ranges are reported.

According to the surgical classification, the medians of detectable MICA, MICB and ULBP-2 levels in DIE, OMA, SUP and control patients are depicted in Fig. 2. MICA (P = 0,015), MICB (P = 0.002) and ULBP-2 (P = 0.045) levels were significantly different between groups. A post-hoc test showed a significant increase in peritoneal MICA and MICB levels in DIE patients with the most severe forms of the disease compared to controls (median, 1.2 pg/mg; range, 0.1–143.5 vs. median, 0.6; range, 0.1–3.5; P< 0.05; median, 5.0 pg/mg; range, 1.4–4702 vs median, 3.6 pg/mg; range, 0.7–20.1; P< 0.01 for MICA and MICB respectively). In addition MICB levels were also significantly increased in SUP patients versus controls (median, 5.6 pg/mg; range, 1.2–35.1 vs. median, 3.6 pg/mg; range, 0.7–20.1; P <0.05). When post-hoc test was performed for ULBP-2 there were significant differences between DIE and SUP patients (median, 0.9 pg/mg; range, 0.2–5.2 vs. median, 0.2 pg/mg; range, 0.1–0.5; P< 0.05).

When all the samples were included in the analysis according to the surgical classification, MICA levels did not show differences between the groups (P = 0.100). However, MICB (P = 0.001) and ULBP-2 (P = 0.026) peritoneal levels were significantly different among groups. The post-hoc test showed for MICB significantly increased values in DIE and SUP patients compared to controls (median, 5.0 pg/mg; range, 1.4–4702 vs. median, 3.4 pg/mg; range, 0.0–20.1; P< 0.01; median, 5.4 pg/mg; range, 0.0–35.1 vs. median, 3.4 pg/mg; range, 0.0–20.1; P< 0.05 for DIE and SUP patients respectively). The same analysis for ULBP-2, showed higher values in the OMA group in comparison to controls (median, 0.0 pg/mg; range, 0.0–1.8 vs. median, 0.0 pg/mg; range, 0.0–4.2; P <0.05) (S2 Table).

### Clinical correlations with peritoneal fluid MICA, MICB and ULBP-2 levels

Clinical, surgical and biological correlations with peritoneal fluid MICA, MICB and ULBP-2 levels in women with endometriosis are expressed in S3 Table.

MICA correlated with MICB (r = 0.466; P <0.001) and ULBP-2 (r = 0.540; P = 0.009). In addition, MICA presented a clinical correlation with dysmenorrhea (r = 0.232; P = 0.029). There was also a positive correlation between MICA and Total rAFS score (r = 0.221; P = 0.031) and with Adhesions rAFS score (r = 0.221; P = 0.031). ULBP-2 levels also correlated with Adhesions rAFS score (r = 0.217; P = 0.034).

MICA correlated not only biologically with other NKG2DLs, but also clinically with pain score and surgically with some surgical findings corresponding to the severity of the disease.

MICA correlations with MICB, ULBP-2, Total rAFS score and Dysmenorrhea are depicted in S1 Fig.

## Discussion

This is the first report on NKG2DLs levels in peritoneal fluid of women with endometriosis. Our study shows that MICB levels are significantly elevated in peritoneal fluid of patients with endometriosis as compared to endometriosis-free women, especially in those patients with the most severe forms of the disease. Concerning peritoneal fluid MICA levels, our results demonstrate higher levels in the endometriosis group than controls, though when considering all the samples (detectable and undetectable values), the differences did not reach statistical significance. The same results could not be obtained for ULBP-2. We found a strong biological correlation between MICA and other NKG2DLs (MICB and ULBP-2). In addition, significant correlations were also found between MICA and clinical characteristics of the disease. High peritoneal fluid levels of MICA were positively correlated with dysmenorrhea, total rAFS score and adhesions rAFS score [34], though these clinical associations had a moderate correlation coefficient.

The strength of this study is based on the originality of the topic and its methodological design: (i) To the best of our knowledge, this is the first study on the assessment of peritoneal fluid NKG2DLs levels in endometriotic patients. Moreover, this study evaluates a large number of peritoneal fluids from patients who were submitted to a gynaecological intervention (121 endometriotic women and 81 controls); (ii) Taking into consideration the heterogeneity of the disease [36], patients were selected with very well-defined clinical phenotypes. The inclusion of patients in the study was performed following a thorough abdominopelvic cavity exploration. All surgical and histological findings were recorded in order to grade the severity of the disease and to distribute each patient according to the former described classification [37]. As shown in previous publications, the wide anatomical heterogenity of endometriosis demands a concrete surgical workup in order to best classify the patients studied [44,45]; (iii) Free-disease patients were clinically and surgically evaluated in the same manner as endometriotic patients. Besides, the wide variety of pathologies present in our control patients exhibits the most common benign conditions in gynaecological patients.

Despite an accurate study design, our results may be subject to certain biases and limitations: (i) though detection rates for MICA and MICB in peritoneal fluids using the ELISA Kit were high (69 and 97% respectively), the rate of detection for ULBP-2 resulted far below from the others (17%). This high rates of undetectable levels, hampers the statistical analysis of data and its interpretation. The rate of undetectable ULBP-2 levels is a result in itself, though the way these undetectable results should be managed for statistical analysis is not clear so far. For this reason, and in order to avoid possible weak points related to the presence of undetectable levels of ULBP-2, MICA and MICB, we carried out two separated statistical assessments: one including and the other excluding the undetectable values [20]. Nevertheless, despite having a low detection rate for ULBP-2, when a detectable value of this ligand was obtained, the likelihood of belonging to an endometriotic patient was significantly higher. These findings lead us to believe an existing role of ULBP-2 in the pathogenesis of the disease; (ii) The high proportion of women with severe endometriosis in our study, does not reflect the real prevalence of disease severity among general population. This selection bias occurs because patients were recruited in our centre, which is a referral centre specialized in the care of grave endometriosis. However, this consideration should not alter the main results of the study; (iii) Despite the control population used in this study display the most common benign gynaecological disorders, it is not clear whether these conditions may modify peritoneal fluid NKG2DLs levels in those patients. The ideal patient used as control should be that without any illness at all. However, nowadays it is hardly feasible to obtain peritoneal fluid from healthy patients. For this reason, and although our results are clear regarding the differences in peritoneal fluid NKG2DLs between endometriotic patients and women without the disease, we believe these data must be interpreted with regard; (iv) The MICA ELISA assay may not equally detect all the different soluble MICA molecules; in fact, more than 60 allelic variants have been described. Our finding that soluble MICA could be detected in endometriotic and control patients suggests that this system was applicable for our cohort of endometriotic patients. As specific allelic variants of MICA exist, we cannot rule out that the differences observed between groups do not reflect various concentrations of different soluble MICA variants. For this reason, special caution should be paid for the use of this ELISA system for widely polymorphic MICA; (v) The ULBP2 ELISA used in this study exhibits some cross reactivity with an other member of the UL16 binding protein family (ULBP), ULBP6. Some ULBP members like ULBP1, 2, 3 and 6 are glycosylphospatidylinositol-anchoring molecules without transmembrane domain that can be proteolytically shed from the surface of cells. One limitation of our study is that there are no strictly ULBP2-specific or ULBP6-specific ELISA available due to the high sequence identity between ULBP2 and ULBP6. Accordingly, studies of soluble ULBP2 likely measured both ULBP2 and ULBP6. Although we cannot exclude that soluble ULBP6 have been detected along with soluble ULBP2 in our patients, the probability for this to occur is expected to be low in our particular clinical situation.

It is nowadays accepted that the immune system actively contributes to the homeostasis of the peritoneal cavity. Women with endometriosis show some kind of immune dysfunction that plays a role in the pathogenesis of the disease [8–11]. Among many other immunological factors, it has been shown that NK lymphocytes display reduced cytotoxicity against endometrial cells [22,26,46]. Thus, it has been suggested that NK cells are implicated in the scavenging of the refluxed endometrial debris in the peritoneum. Some authors have shown that endometriosis surgical treatment does not improve NK cell function [47], which implies that NK dysfunction may be more a cause than a consequence of endometriosis itself. In addition, hormonal treatments such as GnRHa or dienogest, do improve NK cells activity [48–50].

NK cells function is regulated in a highly complex manner by the balance of activating and inhibiting receptors on they surface. It has been stated that an increase in the killer inhibitory receptor (KIR) on NK cells, leads to a reduction of their cytotoxic activity over the endometrial cells [51,52]. Other NK receptors such as natural cytotoxicity receptors have also been studied and showed differences between patients and controls [53].

NKG2D receptor is a major activating receptor on NK cells [54]. NKG2DLs interaction triggers an activating signal and promotes cytotoxic response of the cell expressing the ligand. NKG2DLs are not expressed in most normal healthy cells, but they are frequently overexpressed in infected or transformed cells, acting as a threating signal that enables the immune system to recognize stressed cells [55]. Cell surface expression of NKG2DLs is down-regulated by proteolytic shedding mediated by metalloproteases that come from oxidative stress [30,31,56]. The liberation of soluble forms of the ectodomain of NKG2DLs not only decreases the expression of the ligands on target cells but it also generates the internalization and lysosomal degradation of NKG2D receptor, leading to a paradoxical inhibition of NK cells function. In fact, NKG2DLs shedding has been shown to be a tumour escape mechanism. Elevated NKG2DLs levels have been detected in sera of cancer patients, and correlations among these levels and the stage of the disease or the tumour progression have been demonstrated [27,57,58].

In a similar way to tumours, endometriosis avoids immune surveillance in different manners. In our experience, we believe that the elevation of peritoneal fluid NKG2DLs levels in endometriotic patients is one of the mechanisms of immune dysfunction present in this disorder. In fact, the most severe forms of the disease present higher levels of peritoneal fluid NKG2DLs, leading to a major NK cell dysfunction.

The observed increase in soluble forms of NKG2DLs in peritoneal fluid means a lower expression of these ligands in ectopic endometrial cells surface, heading toward greater evasion from NK cells recognition. Additionally, the rise in soluble NKG2DLs levels further inhibits NK cells cytotoxicity, and consequently NK cell dysfunction becomes more pronounced. According to this rationale, peritoneal invasion and proliferation of ectopic endometrial cells turns out to be more facile when NKG2DLs shedding occurs. The clinical correlations observed in our study reinforce even more our hypothesis.

In conclusion, we demonstrate for first time an increase of peritoneal fluid NKG2DLs in endometriotic patients compared to controls. This study suggests that NKG2DLs shedding is a novel pathway in endometriosis complex pathogenesis that impairs NK cell function. Nevertheless, further studies are needed to determine the genesis of NKG2DLs increase, opening new possible therapeutic targets on this complex disorder [59].

## Supporting Information

S1 Fig Correlation analysis of peritoneal fluid MICA levels with MICB, ULBP and with total rAFS score and dysmenorrhea score. Non-parametric Spearman's correlation tests was used to assess correlations.
(TIFF)

S1 Table NKG2D Ligands qualitative results according to the surgical classification. ^k^ Pearson’s chi-square test; ^f^ Fisher exact test.
(DOCX)

S2 Table Peritoneal NKG2D Ligands levels according to the surgical classification. Analyses were performed both including samples with undetectable levels of NKG2D ligands (i.e. below lower limit of detection of the assay) and without these samples. Results are expressed as median (range). Values are expressed in pg/mg. SUP: superficial peritoneal endometriosis. OMA: Endometrioma. DIE: Deeply infiltrating endometriosis. k. Statistical analysis was performed using Kruskal-Wallis test. Post hoc test were performed using the with Dunn's Multiple Comparison Test. *Significantly different from control women (p <0.05). ** Significantly different from control women (p <0.01). ***Significantly different from SUP (p < 0.05).
(DOCX)

S3 Table Correlation analysis of peritoneal fluid MICA, MICB and ULBP-2 levels and clinical data in women with endometriosis. Note: Pain intensity was evaluated preoperatively using a previously validated 10-cm VAS scale. (26) rAFS: according to The Revised American Fertility Society classification of endometriosis (1). * Only in DIE patients.
(DOCX)
