# Supplementary figures and images for: Soluble Ligands for the NKG2D Receptor Are Released during Endometriosis and Correlate with Disease Severity
Source: PLoS One. 2015 Mar 16;10(3):e0119961. doi: 10.1371/journal.pone.0119961 (PMC4361401; doi:10.1371/journal.pone.0119961)

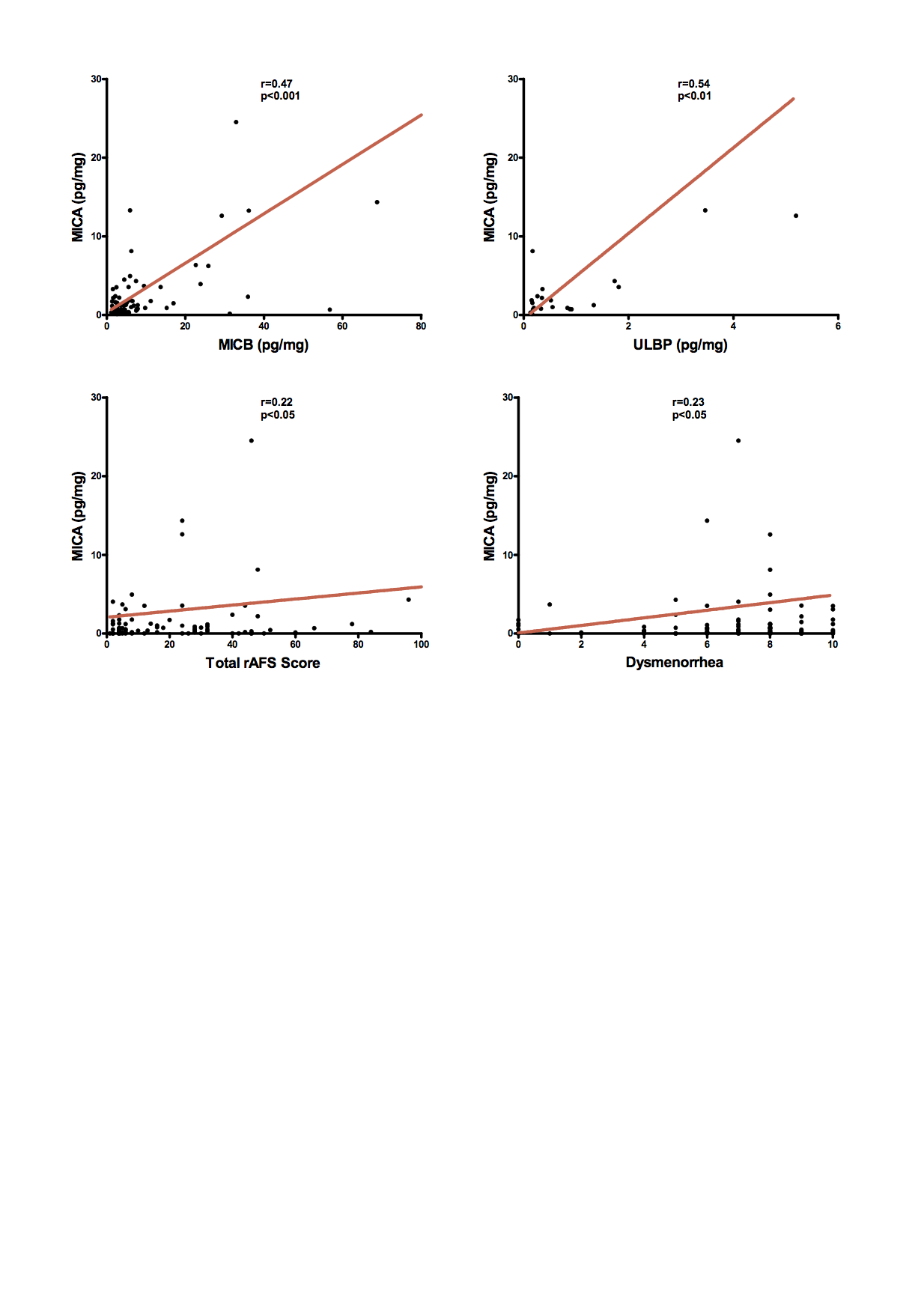

Supplement: S1 Fig — Non-parametric Spearman's correlation tests was used to assess correlations. (TIFF) [file pone.0119961.s001.tiff]
