# Supplementary material for: Soluble Ligands for the NKG2D Receptor Are Released during Endometriosis and Correlate with Disease Severity
Source: PLoS One. 2015 Mar 16;10(3):e0119961. doi: 10.1371/journal.pone.0119961 (PMC4361401; doi:10.1371/journal.pone.0119961)
Supplement: S1 Table — k Pearson’s chi-square test; f Fisher exact test. (DOCX) [file pone.0119961.s002.docx]

| MICA | POSITIVE (n, %) | NEGATIVE (n, %) | p |
| --- | --- | --- | --- |
| SUP (n=41) | 25 (61) | 16 (39) | 0.566 ^k^ |
| OMA (n=32) | 24 (75) | 8 (25) |  |
| DIE (n=48) | 33 (69) | 15 (31) |  |
| Controls (n=81) | 58 (72) | 23 (28) |  |

| MICB | POSITIVE (n, %) | NEGATIVE (n, %) | p |
| --- | --- | --- | --- |
| SUP (n=41) | 39 (95) | 2 (5) | 0.543 ^k^ |
| OMA (n=32) | 31 (97) | 1 (3) |  |
| DIE (n=48) | 48 (1) | 0 (0) |  |
| Controls (n=81) | 78 (96) | 3 (4) |  |

| ULBP-2 | POSITIVE (n, %) | NEGATIVE (n, %) | p |
| --- | --- | --- | --- |
| SUP (n=41) | 7 (17) | 34 (83) | 0.026 ^k^ |
| OMA (n=32) | 10 (31) | 22 (69) |  |
| DIE (n=48) | 10 (21) | 38 (79) |  |
| Controls (n=81) | 7 (9) | 74 (91) |  |

| MICA | POSITIVE (n, %) | NEGATIVE (n, %) | p |
| --- | --- | --- | --- |
| ENDO (n=121) | 82 (68) | 39 (32) | 0.64 ^f^ |
| Controls (n=81) | 58 (72) | 23 (28) |  |

| MICB | POSITIVE (n, %) | NEGATIVE (n, %) | p |
| --- | --- | --- | --- |
| ENDO (n=121) | 118 (97) | 3 (3) | 0.68 ^f^ |
| Controls (n=81) | 78 (96) | 3 (4) |  |

| ULBP-2 | POSITIVE (n, %) | NEGATIVE (n, %) | p |
| --- | --- | --- | --- |
| ENDO (n=121) | 27 (22) | 94 (78) | 0.01 ^f^ |
| Controls (n=81) | 7 (9) | 74 (91) |  |
