# Supplementary material for: Soluble Ligands for the NKG2D Receptor Are Released during Endometriosis and Correlate with Disease Severity
Source: PLoS One. 2015 Mar 16;10(3):e0119961. doi: 10.1371/journal.pone.0119961 (PMC4361401; doi:10.1371/journal.pone.0119961)
Supplement: S2 Table — Analyses were performed both including samples with undetectable levels of NKG2D ligands (i.e. below lower limit of detection of the assay) and without these samples. Results are expressed as median (range). Values are expressed in pg/mg. SUP: superficial peritoneal endometriosis. OMA: Endometrioma. DIE: Deeply infiltrating endometriosis. k. Statistical analysis was performed using Kruskal-Wallis test. Post hoc test were performed using the with Dunn's Multiple Comparison Test. *Significantly different from control women (p <0.05). ** Significantly different from control women (p <0.01). ***Significantly different from SUP (p < 0.05). (DOCX) [file pone.0119961.s003.docx]

|  | DIE | OMA | SUP | Controls | p |
| --- | --- | --- | --- | --- | --- |
| Peritoneal MICA (with) | (n=48)  0.6 (0.0-143.5) | (n=32)  0.6 (0.0-14.3) | (n=41)  0.2 (0.0-4.9) | (n=81)  0.2 (0.0-3.5) | 0.100 ^k^ |
| Peritoneal MICA (without) | (n=33)  1.2 (0.1-143.5)* | (n=24)  0.9 (0.1-14.3) | (n=25)  0.9 (0.1-4.9) | (n=58)  0.6 (0.1-3.5) | 0.015 ^k^ |
| Peritoneal MICB (with) | (n=48)  5.0 (1.4-4702)** | (n=32)  4.5 (0.0-68.7) | (n=41)  5.4 (0.0-35.1)* | (n=81)  3.4 (0.0-20.1) | 0.001 ^k^ |
| Peritoneal MICB (without) | (n=48)  5.0 (1.4-4702)** | (n=31)  4.6 (1.1-68.7) | (n=39)  5.6 (1.2-35.1)* | (n=78)  3.6 (0.7-20.1) | 0.002 ^k^ |
| Peritoneal ULBP-2 (with) | (n=48)  0.0 (0.0-5.2) | (n=32)  0.0 (0.0-1.8)* | (n=41)  0.0 (0.0-0.5) | (n=81)  0.0 (0.0-4.2) | 0.026 ^k^ |
| Peritoneal ULBP-2 (without) | (n=10)  0.9 (0.2-5.2)*** | (n=10)  0.6 (0.1-1.8) | (n=7)  0.2 (0.1-0.5) | (n=7)  0.5 (0.1-4.2) | 0.045 ^k^ |
