# Supplementary material for: Soluble Ligands for the NKG2D Receptor Are Released during Endometriosis and Correlate with Disease Severity
Source: PLoS One. 2015 Mar 16;10(3):e0119961. doi: 10.1371/journal.pone.0119961 (PMC4361401; doi:10.1371/journal.pone.0119961)
Supplement: S3 Table — Note: Pain intensity was evaluated preoperatively using a previously validated 10-cm VAS scale. (26) rAFS: according to The Revised American Fertility Society classification of endometriosis (1). * Only in DIE patients. (DOCX) [file pone.0119961.s004.docx]

| Measurements | MICA | | MICB | | ULBP-2 | |
| --- | --- | --- | --- | --- | --- | --- |
|  | Spearman Rank  Correlation Coefficient (r) | p | Spearman Rank  Correlation Coefficient (r) | p | Spearman Rank  Correlation Coefficient (r) | p |
| Dysmenorrhea | 0.232 | 0.029 | 0.071 | 0.507 | 0.125 | 0.245 |
| Deep dyspareunia | 0,157 | 0.151 | 0.089 | 0.416 | -0.007 | 0.949 |
| Non cyclic chronic pelvic pain | 0.086 | 0.413 | -0.002 | 0.983 | -0.076 | 0.471 |
| Gastrointestinal symptoms | 0.098 | 0.368 | 0.036 | 0.740 | 0.057 | 0.603 |
| Lower urinary tract symptoms | -0.125 | 0.250 | 0.027 | 0.803 | 0.017 | 0.875 |
| Total rAFS score | 0.221 | 0.031 | 0.088 | 0.399 | 0.197 | 0.055 |
| Implants rAFS score | 0.188 | 0.068 | 0.010 | 0.920 | 0.197 | 0.055 |
| Adhesions rAFS score | 0.221 | 0.031 | 0.103 | 0.319 | 0.217 | 0.034 |
| Total number of DIE lesions * | -0.060 | 0.718 | 0.085 | 0.606 | 0.210 | 0.200 |
| MICA | N.A. | N.A. | 0.466 | 0.000 | 0.540 | 0.009 |
| MICB | 0.466 | 0.000 | N.A | N.A | 0.322 | 0.101 |
| ULBP-2 | 0.540 | 0.009 | 0.322 | 0.101 | N.A | N.A |
